# L-GABS: Parametric Modeling of a Generic Active Lumbar Exoskeleton for Ergonomic Impact Assessment

**DOI:** 10.3390/s25051340

**Published:** 2025-02-22

**Authors:** Manuel Pérez-Soto, Javier Marín, José J. Marín

**Affiliations:** 1 IDERGO (Research and Development in Ergonomics), I3A (Instituto de Investigación en Ingeniería de Aragón), University of Zaragoza, C/María de Luna, 3, 50018 Zaragoza, Spain; jmarinbone@unizar.es (J.M.); jjmarin@unizar.es (J.J.M.); 2 Department of Design and Manufacturing Engineering, University of Zaragoza, C/María de Luna, 3, 50018 Zaragoza, Spain

**Keywords:** lumbar active exoskeleton, biomechanics, industrial ergonomics, Forces method, musculoskeletal disorders (MSDs), simulation

## Abstract

Companies increasingly implement exoskeletons in their production lines to reduce musculoskeletal disorders. Studies have been conducted on the general ergonomic effects of exoskeletons in production environments; however, it remains challenging to predict the biomechanical effects these devices may have in specific jobs. This article proposes the parametric modeling of an active lumbar exoskeleton using the Forces ergonomic method, which calculates the ergonomic risk using motion capture in the workplace, considering the internal joint forces. The exoskeleton was studied to model it in the Forces method using a four-phase approach based on experimental observations (Phase 1) and objective data collection via motion capture with inertial sensors and load cells for lifting load movements. From the experimentation the angles of each body segment, the effort perceived by the user, and the activation conditions were obtained (Phase 2). After modeling development (Phase 3), the experimental results regarding the force and risk were evaluated obtaining differences between model and experimental data of 0.971 ± 0.171 kg in chest force and 1.983 ± 0.678% in lumbar risk (Phase 4). This approach provides a tool to evaluate the biomechanical effects of this device in a work task, offering a parametric and direct approximation of the effects prior to implementation.

## 1. Introduction

In industry, where all decisions and measures have an economic and productive influence, simulation and digitalization tools are necessary to simulate the effects these measures have in various company areas. A critical factor to account for is the human factor, particularly in ergonomics, where efficient and well-directed investments are required. In Europe, approximately 60% of work-related health problems are due to musculoskeletal disorders (MSDs) [1], directly economically influencing the company and health system [2]. Intending to alleviate this problem, intelligent ergonomics [3] has emerged, using technological advances to facilitate the work of prevention technicians, whose objective is to reduce the risk of pathologies derived from work.

Prevention technicians have numerous methods to analyze and determine risks in a workplace, with observational methods being the most common in the industry [4]. However, besides requiring considerable time to perform, conventional methods have a high degree of subjectivity. Among the most common are the occupational repetitive action (OCRA) method (cited in ISO 11228-3) [5,6] for repetitive work, the equation from The National Institute for Occupational Safety and Health (NIOSH; cited in ISO 11228-1) [7,8] for manually handling loads, the method in the ISO 11226 standard [9], the rapid entire body assessment (REBA) [10], the rapid upper limb assessment (RULA) [11], or the Ovako working analysis system (OWAS) [12] for evaluating postural loads, and finally, for more general purposes, the European assembly worksheet (EAWS) [13].

The Forces method, presented by Marin et al. [3], reduces the subjectivity of these methods and obtains objective values that do not depend on who conducts the analysis. The Forces method allows the estimation of joint forces through motion capture in the workplace [14]. Estimating Forces risk is based on calculating the internal forces and body movements, as implied by its name. The digitalization of the worker’s movement in the workplace introduces simulation possibilities, anticipating the effects of an ergonomic action, as in the case of simulating the effects on an exoskeleton in the workplace [15].

An exoskeleton is a mobile structure that facilitates movements by applying forces in specific anatomical areas. They can be classified based on various characteristics. Depending on the anatomical area assisted by the forces and torques generated, exoskeletons can be grouped into back-support, upper body, or lower body categories. Based on their design purpose, they can be rehabilitation [16], military [17], or industrial [18] exoskeletons. Depending on the component used to generate the assistive forces and torques, they can be divided into passive [19] and active [20]. Passive exoskeletons provide assistive forces and torques using passive elements, storing or dissipating energy provided by the user through mechanical systems such as springs, elastic materials, or mechanical restrictions. In contrast, active exoskeletons provide assistive forces and torques using actuators controlled by a computer program, depending on sensor information. For active exoskeletons, different control strategies exist, such as direct control, which uses volitional information [21], indirect control, which uses motion information [22], or control based on activity recognition [23].

Finally, depending on the structure and attachments, they can be soft (exosuits), rigid or a combination of both [24]. Soft exoskeletons are based on two garments worn on body segments adjacent to the joint assisted. The assistance is generated by pulling these two body segments together, usually by a dedicated cable or strap. In contrast, rigid exoskeletons are hard and articulated structures that connect actuators to garments worn by the user. An important difference between soft and rigid exoskeletons is the direction of the assistive forces. Soft exoskeletons generate assistive forces parallel to body segments [25], while rigid exoskeletons generate assistive forces perpendicular to body segments [26].

Several studies have been conducted to analyze the influence of implementing different kind of exoskeletons on the risk of MSDs for working personnel [27,28,29] during the workday. These studies highlight the complexity of assessing the biomechanical effects during the execution of specific work tasks. Effectively implanting exoskeletons is a difficult challenge due to the enormous variety of work activities. Determining whether these devices are the optimal solution to ergonomic risks detected in a specific workplace is not straightforward.

One approach to addressing this challenge is integrating the effect of the exoskeleton into ergonomic job assessments. This integration facilitates decision-making using objective criteria. Under this premise, the Forces method establishes an optimal framework to integrate the efforts that exoskeletons exert on the body because the risk scores provided by the Forces method are based on estimating the internal forces and moments. Therefore, Delgado et al. [15] proposed an approach based on the Forces method to consider forces on the body provided by lumbar and upper-body passive exoskeletons.

Given this background, the development of Micro Electro-Mechanical Systems (MEMS) and the accessibility of miniaturized systems related to active exoskeletons, this paper, anticipating future ergonomic challenges, poses the following question: Can the biomechanical effects of an active lumbar exoskeleton be predicted using motion capture at the workstation?

This article aims to answer this question by modeling an active lumbar exoskeleton using the Forces method and estimating the biomechanical effects of its implementation with motion capture. A four-phase method is conducted to design this model based on experimental observations and objective data collection via motion capture and load cells. This method obtains the force curves and activation rules of a commercial active lumbar exoskeleton. The simulated model is compared with the actual values obtained via experimentation regarding forces and risk. This modeling provides an objective predictive tool called: “Lumbar-Generic Active Biomechanics Simulator” (L-GABS), making more efficient ergonomic decisions to implement exoskeleton modeling.

## 2. Materials and Methods

### 2.1. Methodology and Experimentation

This study models the Apogee lumbar active exoskeleton from German Bionic (Augsburg, Germany). In the framework in this paper, modeling an exoskeleton involves defining and mathematically representing the direction, magnitude, and application points of the external forces and torques that the device exerts on the body. This modeling enables calculating the ergonomic risk associated with using the exoskeleton via the Forces method, considering the external forces applied to the user. The four-phase process was designed to achieve the most accurate modeling possible (see Table 1). The phases in this process ensure a detailed exoskeleton study and logical and well-founded model development and evaluation.

Phase 1 aims to understand the operation of the exoskeleton, establish a free body diagram, and define the conditions for applying force and torque. The authors of this article conducted six collaborative testing sessions, each lasting 2 h to conduct Phase 1. The authors specialize in biomedical engineering (PhD), with industrial and mechanical engineering backgrounds.

In each session, one researcher wore the device while the other two took notes. Each researcher used the device in two sessions and participated as an observer in four. The guidelines in the user manual for placing the device and the manufacturer’s illustrative videos were followed [30]. During the sessions, a blackboard was set up with the silhouette of the human body in the sagittal and frontal planes. Directions and force application points were drawn on this silhouette, and after a set of iterations, the resulting free body diagram was obtained. Likewise, an anthropometer (Model 01291 from Lafayette Instrument Company, Lafayette, IN, USA) was employed to measure body dimensions and locate the force application points.

Phase 2 measures the magnitude of the forces (i.e., the torque provided by the device) and determines its relationship with body movement. The participant, familiar with the exoskeleton and the sensations it provides, wore the device during a capture session while the authors provided guidelines to ensure appropriate measurements and supervised data collection. The magnitude of the torque can be regulated via the screen by the user and it depends on spatiotemporal body conditions. A relationship exists between the force and lumbar flexion angle in the passive assistance and between the force and lumbar angular velocity in the active assistance (see Section 2.4).

Phase 3 implements the virtual exoskeleton into the ergonomic method. The signal from all captures was cleaned and adjusted to the same number of points, and a single average curve was generated for each analyzed percentage level. Activation patterns for active assistance were determined, and force curves were generated for modeling. These curves were implemented parametrically in the ergonomic method.

Phase 4 evaluates the exoskeleton modeling. The motion captures for which the values measured by the load cells are available were applied to check modeling errors. To validate the proposed approach two criteria are used: force comparison and risk comparison. In the first part, the force simulated by the virtual exoskeleton is compared with that measured experimentally. In the second part, the estimated risk is compared with the simulated force and the experimentally measured force.

### 2.2. Phase 1: Biomechanical Analysis with the Exoskeleton

**Figure 1:**
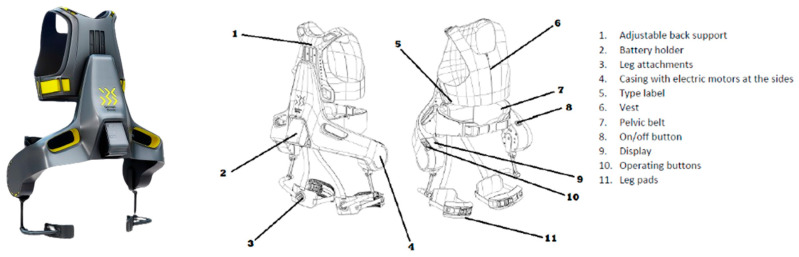
Apogee active exoskeleton diagram (images from Apogee User Manual).

The Apogee exoskeleton from German Bionic is an active exoskeleton that provides lumbar support (Figure 1). Assistive torque is provided by actuators controlled by a computer program that depends on sensor information. The Apogee exoskeleton uses indirect control to activate the actuators, i.e., it relies on motion information. Therefore, when the sensors detect a trunk flexion, the computer activates the actuators, generating a lumbar torque transmitted to the chest and thighs through the garment worn by the user.

Users can combine active and passive assistance in a way they consider appropriate according to their needs, assigning a percentage to each (from 0 to 100 in steps of ten). Selection can be performed via the screen and the buttons located next to the screen in the exoskeleton. The passive assistance of the exoskeleton is presented on the screen with an icon of two linked hands, whereas the active one is depicted on the screen as a flexed arm. Thus, if the passive assistance mode is set to 100% and active to 0%, the user would have the assistance equivalent of a passive exoskeleton; inversely, the user would have a completely active assistance. For any combination where the two are not zero, the user would have a hybrid assistance with both active and passive assistance. Each type of assistance can be described as follow:

- Passive assistance: When selecting 100% passive assistance, the help provided by this configuration is equivalent to using a rigid back-support exoskeleton. During lumbar flexion, the exoskeleton creates a torque resulting in an opening force between the chest and thighs. The torque depends on the user’s lumbar flexion angle ($\theta_{C}$). Owing to its sensorization, unlike passive exoskeletons, the system can work with lumbar flexion concerning a global reference system instead of working with one relative to the lower body.
- Active assistance: The primary purpose of the active control of the exoskeleton is to assist in thorax elevation. Thus, when the ascending speed is greater than the threshold velocity (${\dot{\theta }}_{C}$), the exoskeleton exerts torque to support trunk extension. This torque is related to the user’s lumbar velocity (${\dot{\theta }}_{C}$). In addition, this effect only occurs when ascending from a flexion greater than the active activation angle ($\psi_{A}$).

**Figure 2:**
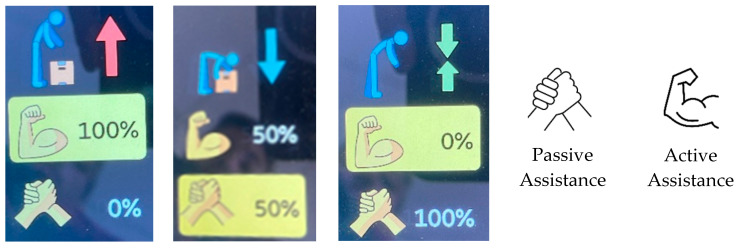
Apogee exoskeleton configuration examples and legend.

Depending on the setting selected by the user (percentage distribution between active and passive), the exoskeleton screen indicates the optimal use of each assistance, whether for lifting (red arrow) or lowering loads (blue arrow) or for prolonged bending work (green arrows) (Figure 2).

**Figure 3:**
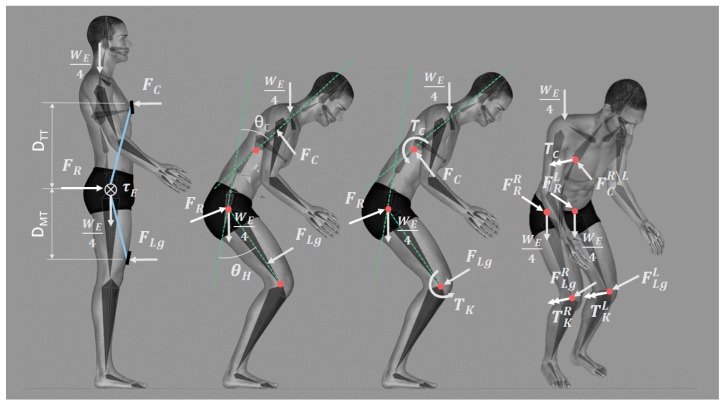
Model diagram forces (right: R, left: L) from Delgado et al. [15] modified for Apogee Exoskeleton.

The free body diagram (Figure 3) of the modeled exoskeleton is based on the study by Delgado et al. [15] and the iterative sessions conducted by the authors (see Section 2.1).

The diagram in Figure 3 represents the efforts of the system through vectors applied at various contact points between the exoskeleton and body. The torque provided by the exoskeleton ($\tau_{E}$) causes the following forces on each side (right, R, or left, L): (1) a force on the chest that aids in recovering the spine’s vertical position ($F_{C}$), (2) a reaction force on each thigh caused by the pressure against the exoskeleton leg pad ($F_{Lg}$) and (3) a force that pushes the pelvis forward, facilitating vertical recovery ($F_{R}$). Additionally, the weight of the exoskeleton distributed between the shoulders and pelvis ($W_{E}$) is considered. These forces are applied to specific points of the body characterized by two dimensions: the distance between the center of the chest pad and trochanter ($D_{TT}$) and the distance between the leg pad and trochanter ($D_{MT}$).

The forces $F_{C}$ and $F_{Lg}$, are transferred to the bones of the human model to solve the equilibrium equations, as presented in Figure 3, at the beginning of the closest bone [3]; the moment generated by the displacement of these forces is considered ($T_{C}$ and $T_{K}$).

Parametric modeling is proposed to allow simple modifications and easily model different but similar commercial exoskeletons in the future. With this parameterization, by modifying the values, other models of active exoskeletons could be modeled straightforwardly, simply by obtaining the values associated with their force curves, determined either experimentally or provided by the manufacturer, as long as the sensations empirically provided are equivalent to those of this device.

### 2.3. Phase 2: Experimentation

**Figure 4:**
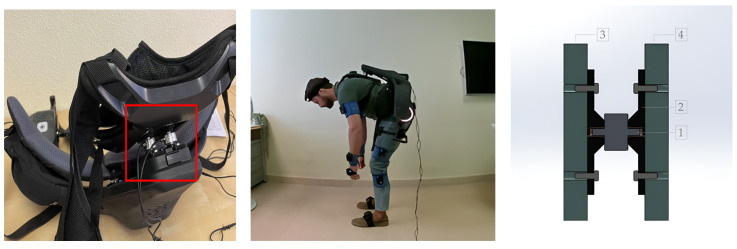
Load cell assembly for force measurement. Experimentation with motion capture and synchronized force measurement. CAD drawing of load cell assembly (1: Load cell, 2: Ad hoc 3D printed part with a threaded insert, 3: Adjustable back support, 4: Casing with electric motors at the side).

For experimental measurements, accessories were designed and 3D printed ad hoc to measure forces using the screw holes in the adjustable back support guides (see Figure 1). These couplings allowed the mounting of two calibrated tensile-compression load cells (Galoce GML668B, Xi’an, China) with a capacity of 50 kg each, connected to a PhidgetBridge 4-Input board (Calgary, AB, Canada) at a sampling frequency of 60 Hz. This coupling and load cell combination was mounted between the exoskeleton structure and the adjustable back support (Figure 4). The sum of the data returned by each load cell provided the force applied to the chest at each frame.

**Figure 5:**
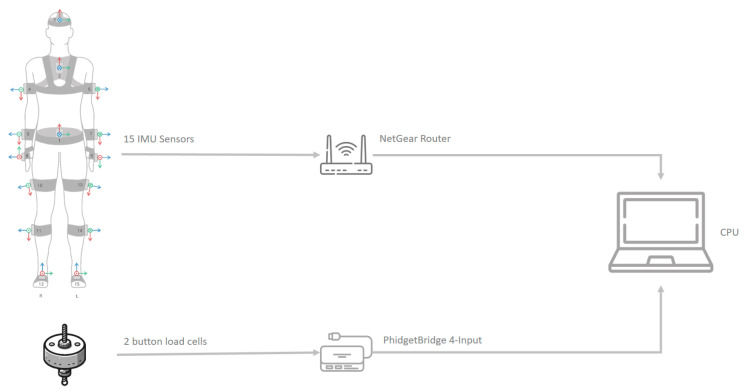
Experimental diagram.

Motion capture was simultaneously performed using inertial measurement units (IMUs) to obtain movement parameters, specifically the x-IMU3 from the manufacturer x-io Technologies (Bristol, UK). This capture system is based on 15 IMUs placed on clothing, capturing movement that is digitized into a 3D human model [3]. This approach provides the value of all biomechanical movement variables synchronized with the measurement of force (Figure 5). The combination of load cells and IMUs data is enough to establish the system’s equilibrium equations.

During Phase 1, the authors analyzed the most comfortable assistance percentages (passive and active) for three tasks: lowering loads, lifting loads, and prolonged bending. The 40% passive assistance was common for lowering and lifting loads. Therefore, to model the device, an intermediate value between maximum assistance and this value is proposed, resulting in three percentages for experimental measurements: 100%, 70%, and 40%. The exoskeleton, instrumented with the load cells and motion capture sensors, was placed on one author (170 cm tall, 70 kg), and various movements were performed as follows:

- The lumbar flexion movement was captured with the exoskeleton at 100%, 70%, and 40% of passive assistance to characterize the passive assistance (active assistance equal to 0%). Three recordings were made for each setting, where the spine was flexed and extended progressively at low speed determined by participant.
- The exoskeleton was configured at 100%, 70%, and 40% of active assistance to characterize the active assistance of the exoskeleton (passive assistance equal to 0%). Three recordings were made for each setting, where the spine was flexed and extended several times, aiming to allow the spine to be carried by the pull provided by the device when ascending. Participants were instructed to ascend at least five times with assistance perception remaining consistent between ascents. Additional ascents were allowed as needed to ensure consistency across all attempts.
- Two captures with hybrid assistance were recorded to obtain data from the passive and active assistances acting in combination: one with full assistance (100% of both active and passive assistances) and another with 50% of each. The recorded motion was spine flexion and extension several times, aiming to be carried by the pull provided by the device when ascending. The participant was instructed to perform random movements to evaluate the precision of the model in unknown motions.

Therefore, 20 recordings were analyzed: nine to characterize the passive assistance, nine for the active assistance, and two to evaluate the model for hybrid assistance. Since the model was obtained using passive and active captures, hybrid ones were used exclusively for model validation.

### 2.4. Phase 3: Model Development and Parameterization

The torque ($\tau_{E}$) provided by the exoskeleton is not constant. It depends on the active ($\eta_{A}$) and passive ($\eta_{P}$) percentages required by the user and on the biomechanical movement variables. As mentioned, passive torque ($\tau_{P}$) is related to the lumbar flexion angle ($\theta_{C}$), and active torque ($\tau_{A}$) is related to the velocity (${\dot{\theta }}_{C}$) measured by the device through the integrated electronics. To maximize the versatility of the parametric model, keypoints (Ξ) are defined for modeling purposes, enabling the simulation of different percentages and the modeling of other exoskeletons in a straightforward manner (see Table A1).

#### 2.4.1. Passive Assistance Model

**Figure 6:**
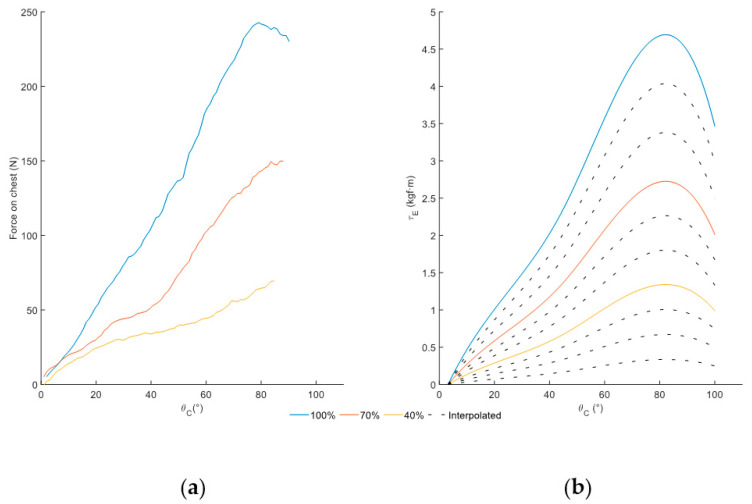
(**a**) Averaged chest force curves experimentally obtained concerning lumbar flexion. (**b**) Modeled passive torque curves on each side.

Figure 6a presents the experimental curve of the passive assistance with levels set to 100%, 70%, and 40%, and with the active assistance set to 0%. These curves relate the force applied to the chest ($F_{C}$) with the lumbar flexion angle ($\theta_{C}$). These curves result from normalizing and averaging the curves from each capture for each percentage. The system provides progressively greater force with lumbar flexion, and greater assistance occurs with the maximum passive degree of assistance ($\eta_{P}$), gradually decreasing with each percentage.

For modeling, the measured force is transformed into passive torque ($\tau_{P}$) on each side, through the distance between the thorax and trochanter ($D_{TT}$), as depicted in Figure 6b. Spline interpolation is employed for passive modeling, as it accurately represents the geometry derived from experimental data (Figure 6a) and preserves a profile similar to passive exoskeletons previously characterized [21,25].

The curves for percentages without experimental data are generated from modeled curves (Figure 6b) once experimental data demonstrate that the behavior across percentages is equivalent. Estimation of the remaining curves is achieved by scaling modeled curves following three steps:

- Estimate the maximum torque for each percentage: The difference between maximum torques in the modeled curves is calculated and evenly distributed across the intermediate percentages.
- Divide the curves into sections using Characteristic Angles (first vector of $\Xi_{P}$): The selected Characteristic Angles were presented by Delgado et al. [15] for modeling lumbar passive exoskeletons.
- Calculate the torque relation (second vector of $\Xi_{P}$): Determine the relationship between the maximum torque and the torque at the characteristic angles for the modeled curves.

Once the maximum torque, the relation between torques, and characteristic angles have been estimated, the five points of the new curve are defined. Thus, when the passive degree of assistance ($\eta_{P}$) is selected, the maximum torque is determined and, as a consequence, the five points characterizing the curve are defined. Spline interpolation is used for each section independently to generate the curves shown in Figure 6b. Therefore, once the angular flexion ($\theta_{C}$) is calculated, the system can estimate the torque assistance by accessing to the corresponding section using lumbar flexion angle ($\theta_{C}$).

#### 2.4.2. Active Assistance Model

**Figure 7:**
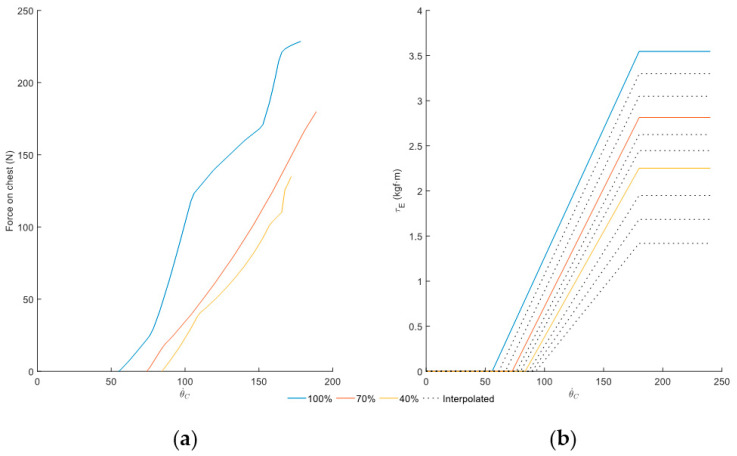
(**a**) Averaged chest force curves obtained experimentally concerning lumbar angular velocity. (**b**) Modeled active torque curves on each side.

Figure 7a presents the experimental curve of the active assistance with percentages configured at 100%, 70%, and 40%, and with the passive assistance percentage configured at 0%. These curves relate the force applied to the chest ($F_{C}$) with the velocity. These curves result from normalizing and averaging the curves from each capture for each percentage. In this case, five peaks per capture were processed, and the ascending part of the cycle is used to represent the curve.

For modeling, the experimental curve Figure 7a is transformed into the curve presented in Figure 7b using the parameters shown in Table A1. Phases 1, 2, and 3 of observation and data analysis allowed the discovery of the relationship between the upward velocity of the trunk and the torque exerted by the device. Likewise, the torque is only applied while ascending and when the ascent velocity exceeds a threshold associated with each percentage (${\dot{\theta }}_{Lim}$), until reaching a maximum value (${\dot{\theta }}_{max}$) beyond which the device provides constant assistance ($\tau_{A}^{max}$) (values are obtained through experimental data and curve shown in Figure 7a). Therefore, to enhance accessibility and versatility, and represent the geometry derived from experimental data, the assistance force of the active assistance ($\tau_{A}$) is parameterized as a series of linear functions defined in sections, depending on the angular velocity of the trunk, which satisfy the following conditions:

(1) $$\tau_{A}({\dot{\theta }}_{C})=\left\{\begin{array}{ccc} 0 & if & {\dot{\theta }}_{C}<{\dot{\theta }}_{Lim}\\ f\left({\dot{\theta }}_{C}\right) & if & {\dot{\theta }}_{Lim}<{\dot{\theta }}_{C}<{\dot{\theta }}_{max}\\ \tau_{A}^{max} & if & {\dot{\theta }}_{C}>{\dot{\theta }}_{max}, \end{array}\right.$$

where $f({\dot{\theta }}_{C})$ denotes the equation of the line that passes through the starting point of the active activation (${\dot{\theta }}_{Lim}$, 0) and the limit at which the maximum assistance is provided (${\dot{\theta }}_{max}$, $\tau_{A}^{max}$).

In an analogous way as in passive model, the curves for percentages without experimental data are obtained from modeled curves. For the active model, the estimation of the remaining curves is achieved by scaling modeled curves following two steps:

- Estimate the maximum torque and velocity thresholds for each percentage (first and second column of $\Xi_{A}$): The difference between the maximum torque and velocity thresholds between the modeled curves are calculated and evenly distributed across the intermediate percentages.
- Obtain the line equation passing through the points (${\dot{\theta }}_{Lim}$, 0) and (${\dot{\theta }}_{max},\tau_{A}^{max}$): The slope and y-intercept of this equation are the third and fourth column of $\Xi_{A}$.

Once the keypoints are estimated, the curves for all percentages can be calculated (dotted lines in Figure 7b). Thus, once the assistance degree ($\eta_{A}$) is selected and the lumbar flexion angle exceeds $\psi_{A}$, the system calculates the assistance using the data from the corresponding row of $\Xi_{A}$. In the first section, the velocity is lower than velocity threshold (second term of corresponding row of $\Xi_{A}$) and no assistance is provided. In the second section, the velocity is between the threshold and ${\dot{\theta }}_{max}$, and the system estimates the torque by solving the linear equation using terms 3 and 4 of the corresponding row of $\Xi_{A}$. Finally, in the third section, the velocity is greater than ${\dot{\theta }}_{max}$ and the torque is the maximum available according to the assistance degree (first term of corresponding row of $\Xi_{A}$).

## 3. Results

To evaluate the model precision, a comparison of forces and risks between virtual exoskeleton and experimental data is performed. For force evaluation, the force on the chest generated by the model is compared with the measured force in the captures. For risk validation, lumbar risk values from the model and experimental data are compared for the captures.

### 3.1. Phase 4: Model Verification

#### 3.1.1. Forces Estimation

Since exoskeleton models assist by creating torque on acetabular joints, the forces must be transmitted to the chest. For validation, the force on the chest estimation is obtained by computing $\tau_{E}=\tau_{P}+\tau_{A}$, and then applying Newton’s second law to obtain Fc from $\tau_{E}$.

**Figure 8:**
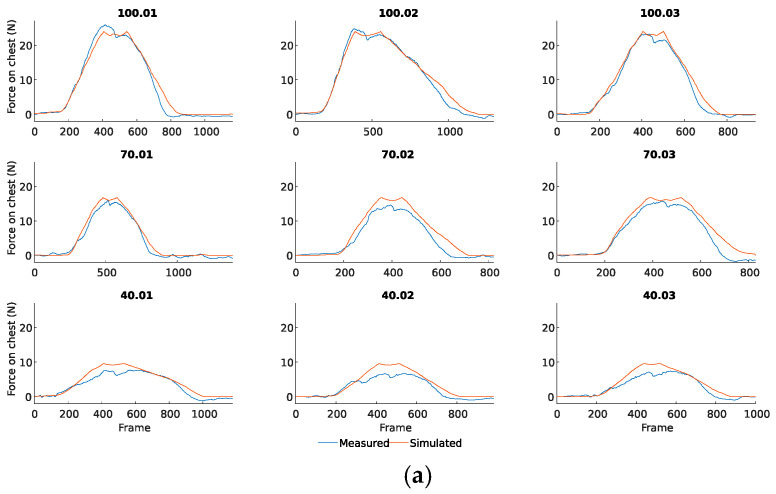

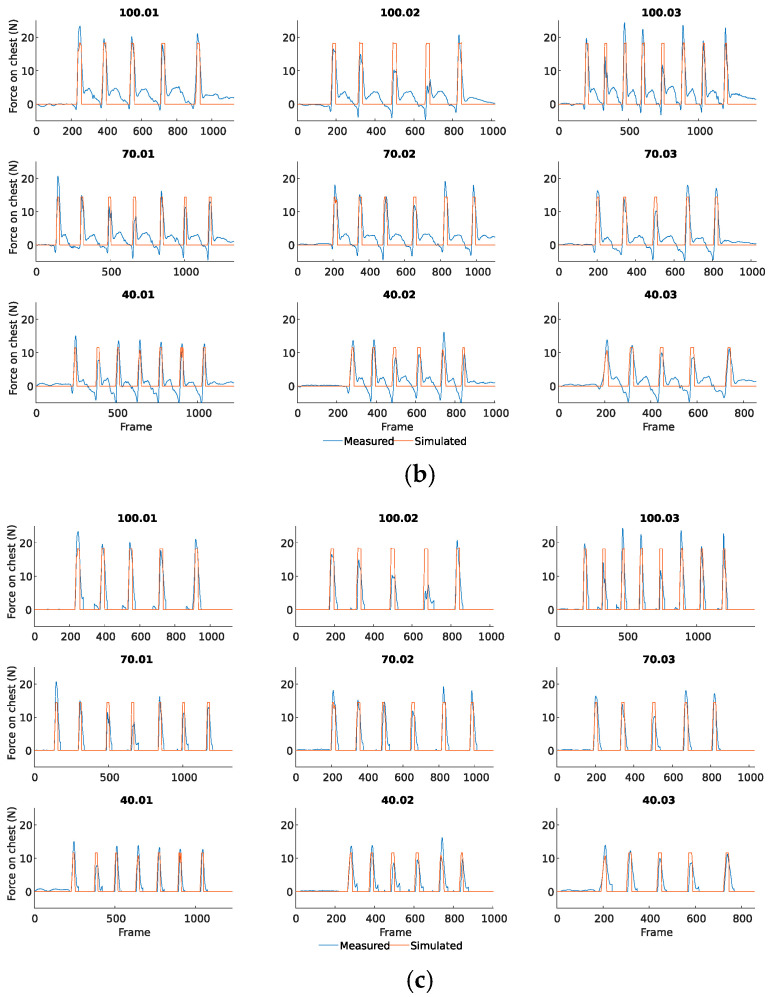
Force measured on the chest compared to the force simulated by the model. (**a**) Passive assistance comparison. (**b**) Active assistance comparison. (**c**) Active Mod. assistance comparison.

Figure 8a compares force terms for the passive assistance, and Figure 8b depicts the forces for the active assistance. Each graph title corresponds to the assistance percentage selected ($\eta_{P}$ in Figure 8a and $\eta_{A}$ in Figure 8b), followed by the capture number for that percentage. For example, capture 100.01 refers to the first capture made with a passive or active percentage of 100%.

The active curve comparison, in descent phases, reveals how the load cells continue to measure the external force that must be exerted to return the chest to its initial position. This external force is applied to the system; thus, the signal is cleaned to reduce errors and compare the values exclusively provided by the exoskeleton (i.e., only during the rising phase; see Figure 8c). Thus, from now on, we will refer to those captures manually modified to only consider load cells data while ascents as Active Mod.

Although only five peaks per capture were used to model the active assistance, all of them were considered for validation. Thus, the model’s behavior in cycles with different assistance sensations reported by the participant is also evaluated.

**Figure 9:**
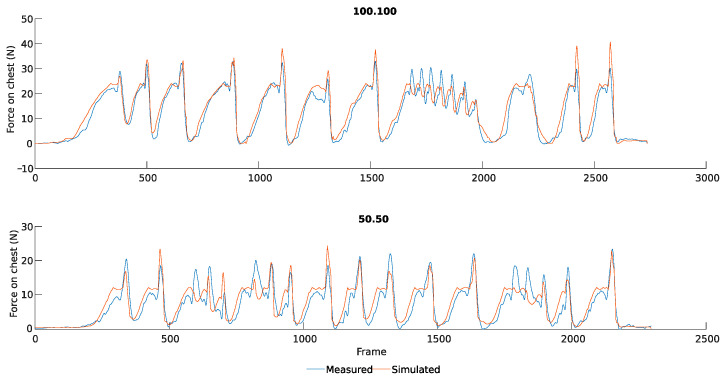
Comparison of the hybrid assistance. Force measured on the chest compared to force simulated by the model.

Once the behavior of passive and active assistance is characterized separately, the results for measurements with a hybrid system are compared (Figure 9). Unlike the previous cases, the title indicates the degree of assistance of the passive and active exoskeleton components. Therefore, a value of 50.50 indicates that the device works in a hybrid model, with active and passive assistance set at 50%. The participant had the freedom in these captures to perform as many flexions and extensions as desired, with the option to stop the ascent halfway in order to test the model’s behavior during half cycles and unknown movements, as can be seen in frames 1500 to 2000 of the 100.100 capture (Figure 9).

Table 2 presents the mean difference values for each capture. The average deviation, considering the passive and Active Mod., is 0.971 kg with a standard deviation of 0.171 kg. The deviation is approximately 0.05 times the maximum force, considering the maximum measured value (about 20 kg). The deviation range is between 0.629 and 2.873 kg.

#### 3.1.2. Risk Estimation

Lumbar joint risks are compared to evaluate how these deviations affect the risk level through the Forces method [3]. The captures where the chest force was measured were used. The ergonomic method was applied, assuming a modeled virtual exoskeleton. In contrast, balance equations were proposed when the force measured on the chest is the reaction to the torque generated by the exoskeleton. Thus, this study measures “virtual” risk and risk using experimental data (Table 3).

The Forces method used in this manuscript is based on motion capture to assess the risk of MSDs resulting from repetitive tasks in industrial environments. The risk of MSDs for various anatomical areas provided by Forces is a percentage representing the ergonomic load of the joint relative to the maximum load obtained through experimentation [3]. A higher percentage indicates that the joint has a higher ergonomic load.

The resulting risk depends on the angle score, angular acceleration score, force score, torque score, and grip score, which are calculated for all postures from the movement data, as indicated in the risk per posture equations described by Marín et al. [3]. Subsequently, to obtain the final risk, the sum of the risks for all postures is calculated, generating a single value called the risk per minute, which summarizes the risk for each joint. Risk per minute represents the repetitive ergonomic risk for each joint and is presented by the Forces method using a color scale for improving the interpretation (Risk < 25% green, 25 ≤ Risk < 40% yellow, Risk ≥ 40% red).

The force score and torque score factors depend on the forces and moments supported by each joint during movement, respectively. Thus, as the exoskeleton is a device that applies external forces to the body, its effect can be estimated by recalculating the kinetics to account for the external forces applied by the exoskeleton on the body. Recalculation, considering the exoskeleton forces and moments, generates different force score and torque score factors for a given capture, while all other factors remain fixed.

Due to the capture protocol in which the cells are tared at the greatest flexion position of the movement, the force that appears between the activation moments of the active assistance is the external force that the body must exert to recover the horizontal position. Therefore, to allow for a fair comparison, when performing the ergonomic calculation, the force must be removed from these intervals so that only the force measured by the gauges during the ascending stages is considered. Otherwise, the approach would assume that the exoskeleton imposes movement restrictions that do not occur in the active assistance, as observed in the results for the hybrid model, for which force exerted by user to recover horizontal position has not been removed from the signal.

Since the captures were made in the laboratory and we are comparing experimental data risk to modeled risk, the order of magnitude and colors provided by the method between different captures are not as important as the differences between the simulated and experimental risks at the same capture. Focusing on differences, results demonstrate a difference in estimation for the passive model at 1.248% ± 0.611%, for the active model at 2.719% ± 0.745%, and for the hybrid model at 18.684%.

## 4. Discussion

This study presents the modeling of the Apogee active lumbar exoskeleton from German Bionic (Augsburg, Germany), enabling the study of its biomechanical effects in a workplace before its implementation and economic expenditure. The presented four-phase method can estimate the forces the exoskeleton would exert on the body. A model was obtained through motion capture, which approximates whether its implementation is ergonomically beneficial. Answering the primary question raised in the introduction: Yes, it is possible to predict the biomechanical effect of an active lumbar exoskeleton using motion capture data measured at the workstation.

### 4.1. Model Application and Industrial Impact

The applications of the presented model are discussed. This model, in combination with motion capture technology and the Forces method, can be a powerful tool for decision-making because it objectifies and quantifies the biomechanical effects of implementing an active exoskeleton without requiring an initial investment in the device to test it in situ.

In addition, the designed parametric model could be used to model other equivalent exoskeletons in the future, considerably improving customization for each simulation and even allowing for the comparison of the effects of different models without having them physically present. Thus, this modeling complements other models of passive exoskeletons [15,31], despite active exoskeletons not having been previously addressed due to the complexity of their electronics and their high weight, among other factors.

Compared to other feasibility studies on industrial exoskeletons, this method is notably less intrusive because it is only necessary to perform motion capture. This capture can determine the effects that the implementation of this system would have on that task. Previous methods to estimate the effects of exoskeletons have been based on electromyography [32] or metabolic expenditure calculations [33], complicating objective data collection; however, this approach offers a simpler alternative.

Modeling this exoskeleton implies the possibility of estimating exoskeleton effects for any task evaluated using the Forces method, regardless of whether the device was worn while the movement was captured. Therefore, this model can be applied in the following two situations:

- A decision criterion (without purchasing the device): In this scenario, modeling becomes a predictive tool to assess the suitability of the exoskeleton without requiring its physical presence at the workstation. This approach allows for evaluating ergonomic improvements based on the model, making it easier to screen potential options. The process involves three steps: (1) capturing the movement in the workplace, (2) applying the Forces method to determine the initial risk, and (3) reapplying the Forces method using the same captured movement, this time accounting for the effects of the exoskeleton. Two evaluations can be performed for the same task: one without considering the device assistance and another that includes the effects of the exoskeleton on the body.
- An evaluation of the effects (with the device): Exoskeletons influence the worker’s movements; thus, the user must become accustomed to wearing the device. It may be helpful to reevaluate the movement after integrating the exoskeleton into the workplace to assess its final effects. This evaluation can serve as a crucial argument to continue promoting the device’s use and can help convince critical stakeholders involved in industrial prevention efforts. The process involves four steps: (1) capturing the initial movement in the workplace, (2) applying the Forces method to determine the initial risk, (3) capturing the movement again after several weeks of using the exoskeleton, and (4) applying the Forces method to this new capture, considering the exoskeleton effects. In this scenario, two evaluations are conducted for the same task: one without considering the device assistance and another incorporating the new movement patterns and effects of the exoskeleton.

This second scenario, in which the device is available, opens a wide range of future studies, such as the effect of long-term use of the exoskeleton and potential human adaptive changes, as well as relating biomechanical benefits to user experience (comfort, acceptance, and satisfaction), which are key factors for successful exoskeleton implementation. It could also involve integration with other ergonomic tools, such as markerless motion capture applications.

### 4.2. Modeling Method: Limitations and Considerations

The four-phase method can be extended to other modeling studies, providing a structured and well-founded approach to modeling. However, the proposed model has the following limitations and simplifications, which should be considered when replicating this method:

- Access to system electronics: Direct access to the exoskeleton’s electronics would greatly benefit the modeling process. Collaboration with the manufacturer could enhance the precision of measurements, estimations, and calculations, leading to a more accurate model. In addition, patterns activation would be completely defined reducing uncertainties in the variables relationships.
- Separation of passive and active modeling: Passive and active assistances were modeled separately. The coexistence of two exoskeletons in a single system was assumed, given the device’s performance and specifications. However, uncertainties in each model could accumulate when performing hybrid simulations.
- Hysteresis effect: During the analysis, the hysteresis effect, where a device applies different forces when the torso moves up versus down, was negligible. Thus, a single curve was applied for upward and downward movements. This work assumes that the electronic system includes a controller that compensates for energy loss caused by this effect.
- Beyond biomechanical factors: Implementing exoskeletons in the workplace addresses more than just biomechanical considerations. Worker comfort [34], temperature [35], and social well-being [36] also play essential roles, as do productivity-related factors. Biomechanical estimations are helpful for screening but do not account for these other variables, which may require further study to determine the overall effectiveness.
- Experimental synchronized data: Another potential source of error is the synchronization between load cells and IMUs. To address this issue, a source code was implemented to simultaneously launch IMUs and load cell measurement. When the capture finished, if the data were inconsistent an error message appeared, and the capture was repeated.
- Dissipation effects: One limitation of the model is that it considers the torque exerted by the exoskeleton as a single force applied to the user’s body, without accounting for any load distribution along the contact surface.

Regarding the limitations, the curves were obtained without direct access to the system’s electronics, introducing uncertainty and potential protocol and sensor calibration errors. While the error associated with the sensorization is relatively small—approximately 50 g per load cell—compared with the measured forces, the body’s inertial forces are considered the primary source of error. Therefore, given the lack of a definitive “gold standard” in these cases, it is not possible to assert that the experimental data are more precise than the modeled results or vice versa.

### 4.3. Model Result Interpretation

Concerning the discussion of numerical results (Phase 4), considering the context of industrial ergonomics where this modeling can be applied, the margin of error is quite reasonable. The virtual model achieved approximately 95% precision compared with experimental measurements, with the average difference per capture ranging between 0.629 and 2.873 kg. Given the ergonomic application for which this model was designed, this variation is deemed acceptable.

In the passive assistance model, the discrepancy became larger as the measurements approached zero. However, as indicated in Table 2, the results remained reasonable, with the worst-case mean deviation for the entire capture being 1.53 kg.

In the active assistance model, higher deviation values were observed due in part to the fact that multiple cycles occurred per capture. Additionally, during the transition phase between cycles, a force must be applied to return to the initial horizontal position. The sensors measure this force but do not include it in the model, leading to a considerable increase in the average deviation throughout the capture. Despite this, the highest mean difference in the active model without correction was 2.63 kg.

To correct this discrepancy and enable a fair comparison between the virtual model and the actual force exerted by the exoskeleton on the body (as opposed to the reverse), the signal during these transitional phases is filtered out, excluding the force measured during the lowering stages of the capture. This adjustment significantly improved the results, with the worst-case simulation exhibiting a deviation of 1.236 kg.

In the hybrid comparison, the virtual system provided reasonable values, even in movements with notable inertia. This finding is not problematic, considering the uncertainties introduced by the experimentation process and the forces required for lumbar flexion. However, deviations of 2.391 and 2.114 kg were observed for these movements, respectively.

The risk associated with active and hybrid captures is considerably higher than that estimated for passive captures. In passive captures, the movement consists of a single flexion-extension at low velocity, as defined by the user. In contrast, active and hybrid captures involve at least five flexion-extension cycles. Furthermore, during the ascent in active captures, the participant was required to allow the force exerted by the exoskeleton to guide their movement without resisting, resulting in higher velocities and accelerations. The Forces method estimates the risk based on the repetition of the capture throughout the workday. Therefore, the risks for active captures are calculated assuming five times more cycles than for passive captures, along with higher velocities and accelerations.

Similarly, higher velocities, accelerations, and number of cycles can lead to higher risks, even with higher assistance as can be seen in captures 100.03 (8 cycles) or 70.02 (7 cycles) where risks are higher than those obtained with 40% assistance. However, to evaluate the precision of the model, the focus is on the comparison between the simulated risks and those obtained from experimental data for the same capture.

Comparing risks for the same capture, the passive model exhibited a deviation of 1.248% in risk estimation, with a deviation range of 0.611%, maintaining consistent values across all captures within the range. For the active model, risks were calculated exclusively using the modified signal values to achieve the highest possible realism while avoiding the inclusion of the force required to return to the initial position. These adjusted values obtained a median deviation of 2.719% and an average deviation of 0.745%.

High deviations were observed in the hybrid captures. This discrepancy between the model and experimental data in hybrid captures lies in the force exerted by the user on the system. Analyzing the data from frames 1500 to 2000 in capture 100.100, where the participant ascended intermittently, the measured force is higher than the simulated force due to inertial forces measured by the load cells. This force deviation should directly affect risk estimation, as is mentioned in Section 3.1.2, through force and torque score.

In addition, the randomness of the proposed movement prevents effective error reduction in the signal as in Active Mod. captures. However, this situation allows for empirical validation, confirming that the modifications for the active case are coherent and effective in accurately assessing the real risks associated with the movement.

A powerful tool for industrial ergonomics research was developed, considering all of these factors. The tool can objectively simulate the biomechanical effects of an active lumbar exoskeleton in a workplace setting by modeling a commercial exoskeleton. This simulation is achieved with minimal intrusiveness because only the motion capture of the task is required once the model is digitized.

## 5. Conclusions

A commercial active exoskeleton was modeled, allowing the simulation of the forces generated by the device on the human body through motion capture. This approach enables the inference of the biomechanical consequences of its use during specific tasks.

The force values generated by the proposed digital model (virtual exoskeleton) were compared with the measurements obtained from load sensors during experimentation to validate the model. Additionally, the translation of these forces into ergonomic risk was assessed using the Forces method for various recorded tasks.

Given the work context and the purpose of the model, which is to support industrial ergonomic studies, a powerful tool was developed despite the simplifications in the modeling process. This tool can assist prevention specialists in decision-making to reduce the risk of MSDs by providing objective data.

## Figures and Tables

**Table 1:** Research phases and techniques for modeling the active lumbar exoskeleton.

Phase	Objective	Research Technique	Description
1. Biomechanical analysis with the exoskeleton	Characterize and understand how the exoskeleton works.	Experimental observation and objective measurement.	Place the device, assess its effect, and consult the user manual. Establish the free body diagram and the patterns of force and torque application. Measure dimensions and locate the points of application of forces.
2. Experimentation	Forces magnitude and their relationship with the movement of the body.	Objective measurement.	Weigh the exoskeleton. Measure the force applied to the body using load cells and record lumbar flexion with motion capture simultaneously.
3. Model Development and Parameterization	Model implementation in the ergonomic method.	Data processing and software development.	Process the experimental data, obtain force curves and activation patterns. Implement the modeling in the virtual environment in a parametric manner.
4. Model Verification	Model evaluation at force and ergonomic risk level.	Comparison.	Compare forces and risk obtained by the modeling with the experimental results.

**Table 2:** Deviation between experimental and virtual data for passive, active, Active Mod. and hybrid models.

**MEAN DIFF. CAPTURE [KG]**	**100.01**	**100.02**	**100.03**	**70.01**	**70.02**	**70.03**	**40.01**	**40.02**	**40.03**
**PASSIVE**	0.886	0.833	0.854	0.869	1.039	0.936	0.629	1.536	1.321
**ACTIVE**	2.237	2.213	2.636	1.160	1.490	1.461	1.993	1.941	1.281
**ACTIVE MOD.**	0.826	1.236	1.179	0.815	1.007	0.891	0.997	0.927	0.690
**MEAN DIFF. CAPTURE [KG]**	**100.100**	**50.50**							
**HYBRID**	2.391	2.114							

**Table 3:** Risk comparison between simulation and experimental data (Risk < 25% green, 25 ≤ Risk < 40% yellow, Risk ≥ 40% red).

Passive Assistance		Active Assistance		Hybrid Assistance	
Capture	Lumbar Risk	Capture	Lumbar Risk	Capture	Lumbar Risk
S100.01	7.365%	S100.01	91.156%	S100.100	24.405%
ED100.01	7.579%	ED100.01	88.912%	ED100.100	40.150%
S100.02	8.558%	S100.02	97.563%	S50.50	26.987%
ED100.02	8.904%	ED100.02	101.002%	ED50.50	48.609%
S100.03	7.844%	S100.03	106.611%		
ED100.03	8.693%	ED100.03	106.085%		
S70.01	8.900%	S70.01	98.836%		
ED70.01	9.732%	ED70.01	101.678%		
S70.02	11.925%	S70.02	109.571%		
ED70.02	14.028%	ED70.02	111.917%		
S70.03	14.490%	S70.03	99.169%		
ED70.03	16.436%	ED70.03	101.722%		
S40.01	18.645%	S40.01	108.177%		
ED40.01	20.217%	ED40.01	111.662%		
S40.02	15.022%	S40.02	106.177%		
ED40.02	16.803%	ED40.02	110.641%		
S40.03	15.244%	S40.03	90.864%		
ED40.03	16.835%	ED40.03	93.438%		

Simulated (S), Experimental Data (ED).

## Data Availability

Data are contained within the article.

## Appendix A

The parameters used for the model are shown in Table A1.

**Table A1:** Parameters used in the model.

Nomenclature	Description	Exoskeleton Value
$W_{E}$	Weight of the exoskeleton with all its components and considered due to carrying the system	8.676 kg
$D_{TT}$	Distance between the center of the chest pad and the trochanter for a 170 cm person. Relationship between $D_{TT}$ and spine length	38 cm 0.72
$D_{MT}$	Distance between the thigh pad and the trochanter for a 170 cm person. Relationship between $D_{MT}$ and femur length	18 cm 0.43
**Passive Model Parameters**		
$\eta_{P}$	Assistance degree (as per one) of the exoskeleton passive assistance	0.0–1
$\Xi_{P}$	Keypoints characterization in passive curve modeling First vector: Characteristic angles Second vector: Relationship between maximum torque and torque the provided in that section (as per one)	[10, 20, 45, 80, 100] [0.100, 0.215, 0.503, 1.000, 0.741]
$\tau_{P}^{max}$	Maximum passive torque achieved by the exoskeleton	4.68 kg·m
**Active Model Parameters**		
$\eta_{A}$	Assistance degree (as per one) of the exoskeleton active assistance	0.0–1
$\psi_{A}$	Lumbar flexion angle from which the active system provides assistance (always depending on velocity)	20
$\Xi_{A}$ **	Keypoints characterization in active curve modeling	[0, 0, 0, 0] 0% [1.419, 92.628, 0.016, −1.504] 10% [1.596, 89.280, 0.018, −1.571] 20% [1.951, 86.490, 0.021, −1.804] 30% [2.252, 83.700, 0.023, −1.958] 40% [2.447, 79.794, 0.024, −1.949] 50% [2.624, 76.279, 0.025, −1.930] 60% [2.814, 72.601, 0.026, −1.902] 70% [3.050, 66.960, 0.027, −1.807] 80% [3.298, 61.380, 0.028, −1.707] 90% [3.547, 55.800, 0.029, −1.593] 100%
${\dot{\theta }}_{max}$	Velocity from which the maximum assistance of the active exoskeleton is provided	180°/s

** Matrix structure ($\tau_{A}^{max}$, ${\dot{\theta }}_{C}$, *m*, *n*) where *m* and *n* represent the slope and y-intercept of the line equation passing through the points (${\dot{\theta }}_{Lim}$, 0) and (${\dot{\theta }}_{max},\;\tau_{A}^{max}$).
